# An Evaluation of the Comprehensibility Levels of Ophthalmology Surgical Consent Forms

**DOI:** 10.7759/cureus.16639

**Published:** 2021-07-26

**Authors:** Ibrahim Ethem Ay, Mustafa Doğan

**Affiliations:** 1 Ophthalmology, Sandikli State Hospital, Afyonkarahisar, TUR; 2 Ophthalmology, Afyonkarahisar University of Health Sciences, Afyonkarahisar, TUR

**Keywords:** informed consent, comprehension, readability, ophthalmology, medical law

## Abstract

Background/Aim

This study aimed to evaluate the comprehensibility of the consent forms used for interventional procedures in the ophthalmology clinic of a university hospital and to determine which texts could be read according to patient age and education level.

Materials and methods

Forty separate consent forms used as the standard for various interventional procedures in the ophthalmology department of a university hospital were evaluated. The comprehensibility formulas used were developed for the Turkish language by Ateşman and Bezirci-Yilmaz.

Results

As a result of the evaluation of the consent forms in this study, a mean of 55.6±5.73 points was obtained according to the Ateşman comprehensibility index, and this value was found to correspond to being understood by eleventh and twelfth-grade school students. According to the Bezirci-Yilmaz comprehensibility index, the mean points of the consent forms were 10.05±2, which corresponded to a level that could be understood by 10th and 11th-grade students.

Conclusion

The comprehensibility level of the consent forms given to patients was found to be low in this study, which was similar to the findings of previous studies in the literature. When preparing informed consent forms, the education level of the country must be taken into consideration.

## Introduction

The term “comprehensibility” is used to define whether a text can be understood by the reader. Nowadays, the comprehensibility of a text can be measured objectively with mathematical formulas. Various methods have been used to determine the comprehensibility rates of scientific texts [1]. In 1952, the Gunning-Fog value was introduced to determine for which age group a text was suitable by examining the length of words in a sentence and how many words were used in a sentence [2]. The Flesch-Kincaid value provided a comprehensibility criterion according to the education level of the reader, in addition to stating which age group could read a text [3].

To evaluate the comprehensibility of texts, parameters are used such as how many words are in the sentences, how many syllables form the high-frequency words used, and the frequency of using abstract words and technical terms. Thus, more than 40 different formulas have been developed, which are accepted as comprehensibility criteria [4]. Consent forms for interventional procedures are published on the website of the Turkish Ophthalmology Association (www.todnet.org), which can be used by ophthalmologists who are members of the association, and they are encouraged to do so. The ophthalmology department of Afyonkarahisar Health Sciences University (AHSU) started to use these forms with some small changes made for adaptation to the population of the province. The aim of this study was to evaluate the comprehensibility of the consent forms used for interventional procedures in the ophthalmology clinic of a university hospital (AHSU) and to determine which texts could be read according to patient age and education level.

## Materials and methods

In Turkey, consent forms are used according to the preference of each hospital or each surgeon. These forms may be standard or personalized by evaluating examination findings. In this study, draft consent forms recommended by the Turkish Ophthalmology Society were used, and 40 separate consent forms used for various interventional procedures in the ophthalmology department of a tertiary-level university hospital were evaluated.

Each form was uploaded to the Microsoft Notepad program (Microsoft Corporation, Redmond, WA). The titles of the forms were removed to prevent any incorrect effect on the comprehensibility level and then the forms were evaluated in the context of the comprehensibility formulas developed for the Turkish language by Ateşman, and Bezirci-Yilmaz. When performing these procedures, the computer software developed by Bezirci-Yilmaz was used [5-6]. In addition, the number of medical terms used on the forms was recorded, the ratio of medical terms to total words was calculated, and this ratio was stated as a percentage.

Two different comprehensibility formulas were used in this study. The Ateşman comprehensibility formula was applied as:

198.825 - 40.175 x word length (total syllables / total words) - 2.610 x sentence length (total words/ total sentences).

The numerical value obtained determined the comprehensibility level of the text according to the education level of the reader. As a result of the analysis made using the Ateşman comprehensibility formula, a comprehensibility value was obtained in the range of 0-100. Higher values approaching 100 indicate easier comprehensibility of the text and values approaching zero indicate greater difficulty. In the formula developed by Bezirci-Yilmaz, a graph is formulated according to the number of words in a text, the number of syllables in the words, and the distribution of the number of syllables in the words (Table 1).

**Table 1:** The relationship between the Ateşman comprehensibility index and the comprehensibility level

Index	Comprehensibility Level
90 – 100	Can be easily understood by students in 4th grade and below
80 – 89	Can be easily understood by students in 5th and 6th grade
70 – 79	Can be easily understood by students in 7th and 8th grade
60 – 69	Can be easily understood by students in 9th and 10th grade
50 – 59	Can be easily understood by students in 11th and 12th grade
40 – 49	Can be easily understood by students in 13th or 15th grade (undergraduates)
30 – 39	Can be easily understood by university graduates
≤ 29	Can be easily understood by university postgraduates

Bezirci-Yılmaz Formula = √MWC x ((S3 x 0.84) + (S4x1.5) + (S5x3.5) + (S6x26.25)) where MWC: mean word count; S3: mean number of words with three syllables; S4: mean number of words with four syllables; S5: mean number of words with five syllables; S6: mean number of words with ≥ six syllables 

The results obtained from this formula show the level of comprehensibility of the text according to the education system in Turkey (Table 2).

**Table 2:** The relationship between the Bezirci-Yilmaz comprehensibility index and the comprehensibility level

Index	Education Level
1–8	Primary school
9–12	High school
12–16	Undergraduate
16+	Postgraduate

## Results

As a result of the evaluation of the consent forms in this study, mean 55.6±5.73 points were obtained according to the Ateşman comprehensibility index, and this value was found to correspond to being understood by eleventh and twelfth-grade school students. According to the Bezirci-Yilmaz comprehensibility index, the mean points of the consent forms were 10.05±2, which corresponded to a level that could be understood by tenth and 11th-grade students.

When all the forms were evaluated together according to the Ateşman criteria, 29 consent forms were found to be comprehensible with education at the level of thirteenth, fourteenth, and fifteenth grades. The other forms were determined to be comprehensible at the high school level of education (grades 9, 10, 11, 12). No form was determined to be comprehensible at the level of primary school education.

According to the Bezirci-Yilmaz criteria, only one form was seen to be comprehensible at the level of primary school education, 16 forms were determined to be comprehensible with a high school level of education (grades 9, 10, 11, 12), 22 forms at undergraduate level (grades 13, 14, 15), and one form at postgraduate level (grade 16+) (Table 3).

**Table 3:** The distribution of the comprehensibility levels of the consent forms according to the Ateşman index and the Bezirci-Yılmaz index

	Primary school (4, 5, 6, 7, 8)	High school (9, 10, 11, 12)	Undergraduate (13, 14, 15)	Postgraduate (+16)
Ateşman index	0	11	29	0
Bezirci -Yilmaz index	1	16	22	1

## Discussion

The first serious comprehensibility formula was devised by Fresch in 1948. In the calculation of the Fresch Reading Ease Score (FRES), the ratios of words to sentences and syllables to words are used [7]. The Simple Measurement of Gobbledygook (SMOG) was designed as a comprehensibility criterion by Mclaughlin in 1969. The determination of the SMOG value includes the calculation of the number of words of three or more syllables, and sections are evaluated of at least 10 sentences from the beginning, middle, and end of the text. Following the application of mathematical formulas, a comprehensibility value is provided corresponding to the American education system [8].

The Automatic Readable Index (ARI) is a formula devised in 1967 by Smith and Senter to standardize the comprehensibility of technical documents in the US army. The mean length of words in the document is determined, and how many letters comprise these words is calculated. The result indicates to which age group the text is targetted [9].

In Turkey, the Ateşman formula was first developed in 1997. According to this formula, the mean sentence length in Turkish is nine to 10 words, and the mean length of the words is 2.6 syllables. When the required evaluation is made with the mathematical formula determined by Ateşman in the light of these data, it is possible to determine for which level of education the text is suitable [5].

In 2010, a new comprehensibility formula for the Turkish language was recommended by Bezirci-Yilmaz. The total number of sentences in the text, number of words, number of syllables, number of letters, and number of words with more than four syllables are used in this formula. In addition, by determining the mean number of syllables of the words in the text, graphs are produced of the distribution of words according to the number of syllables. The data obtained provide a comprehensibility value of the text according to the educational level of the reader [6].

In a 2019 study by Ebem et al., 90 separate intramuscular and intravenous consent forms were evaluated using the formulas developed by Ateşman and Bezirci-Yilmaz, and the results showed that the comprehensibility level of the intramuscular and intravenous consent forms used by the hospital was extremely low [10]. Ebem et al. found that a comprehensibility value of 56 points for Ateşman corresponded to eleventh and twelfth-grade students. That finding was consistent with the current study results. In the same study, 9.43 points in the Bezirci-Yilmaz comprehensibility index were found to correspond to grades 10 and 11. Although this was consistent with the current study, the comprehensibility level according to the Bezirci-Yilmaz index was found to be lower.

In the current study, medical terms in the text were observed at the rate of 3.7%. When it is considered that consent forms usually consist of 1200-1400 words, this shows that 44-52 words on each consent form are medical terms. This feature can make it more difficult to read the form. Ebem et al. reported a lower rate of 2.6% of medical terms in the intramuscular and intravenous consent forms.

In a study of the comprehensibility of the consent forms used in a Skin Diseases and Leprosy Training and Research Centre in Tehran, the Flesch-Kincaid and Gunning-Fog indexes were used, and as a result of the evaluations made with both indexes, the consent forms were determined to be at a level that would be comprehensible on average by an eleventh-grade student. This was interpreted by the researchers as an extremely low level of comprehensibility of the consent forms [11].

In research conducted by the American Medical Association and the National Health Institute, seven separate formulas were used, including the Flesch Kincaid. The results determined that the consent forms used for invasive procedures were of a comprehensibility level corresponding on average to an educational level of fifteenth grade. The average educational level of adults in the USA was determined to be eighth grade, and thus it was stated that the comprehensibility level of the consent forms used for invasive procedures was extremely low. The American Medical Association and the National Health Institute recommended that consent forms used for invasive procedures in the USA should be at a level that could be read at an education level corresponding to the sixth grade [12].

Shuba et al. conducted a study of the informed consent forms signed by oncology patients receiving radiotherapy and evaluated 113 forms used in 89 separate centers. The results demonstrated that 100 forms could be read by individuals with a level of education of tenth to fourteenth grade. Four forms were found to be comprehensible at the eighth-grade level and four forms at the sixth-grade level. It was emphasized in that study that consent forms should be prepared at a more comprehensible level [13].

It is a legal and ethical requirement that a patient knows what will be done to his body and that an informed consent form is obtained before any interventional procedure. It is also necessary that the person who is to give consent has been appropriately informed and can consent to the intervention in question. For the patient to be appropriately informed, he must be informed about why the intervention is necessary, how it will be done, the benefit to the patient, potential complications must be explained in detail, and alternative opinions of the treatment must be given [14].

The obtaining of signed informed consent is accepted as a precondition, both legally and ethically [15]. Providing the patient with information verbally has the advantage that it can be adjusted according to age and education level. However, as written consent forms are issued as standard in most clinics and cannot be changed according to each case, they should be prepared at a high level of comprehensibility [16]. If only verbal consent is obtained or the written consent is not sufficiently clear and understandable, the person providing the information may be legally responsible and the consent may not be valid [17].

In a study by Boztaş et al. on the comprehensibility of anesthesia consent forms in Turkey, the rate of medical terms in the forms was determined to be 4%, and the custom of reading and signing the forms was reported to be extremely low [18]. In a study conducted in Turkey in 2008, the intelligibility level of consent forms for cataract surgery was questioned by applying a questionnaire to the patients, and the level of intelligibility was found to be quite low. In our study, consent forms were evaluated with the readability index [19].

When evaluating the comprehensibility of the consent forms in this study, factors such as the length of the sentences in the text, the number of words, and the number of syllables in the words were taken into consideration. Before an operation, the anxiety level of patients can be expected to be high, and this can affect the comprehensibility of the text. In addition, the nature and size of the font used in the text can also affect comprehensibility. It can be concluded that it would be useful to prepare new informed consent forms taking into consideration education level, age, gender, other demographic factors, and the visual acuity of the patient. The average education level in Turkey is 6.51 years [20]. Consent forms must be legible for lower education levels. For a patient with sight problems who is to go undergo an interventional procedure, the comprehensibility of the text will be even more difficult. Consent forms that are more clear and can be understood should be issued to these cases. There remains a need for further studies in this area.

## Conclusions

The data obtained in this study demonstrated that the comprehensibility of the consent forms issued to patients in the ophthalmology department of a tertiary-level university hospital was at an extremely low level. Although there are no studies in the literature related to the mean age of the population who require an ophthalmology interventional procedure, when it is considered that the frequency of diseases such as cataract, senile macular degeneration, glaucoma, and diabetic retinopathy increases with age, the patient group requiring interventional procedures can be expected to be above the average age of the Turkish population. The educational level of patients of advanced age in Turkey can be anticipated to be lower than average. Therefore, informed consent forms prepared for interventional diseases in the field of ophthalmology should be comprehensible at least at the level of primary education. The data obtained for the consent forms examined in this study were seen to be at a low level of comprehensibility, similar to the findings of previous studies in the literature. When preparing informed consent forms, the education level of the country must be taken into consideration. In this way, the limits of legal responsibility of healthcare workers can be better defined.
